# Analgesic, neuropharmacological, anti-diarrheal, and cytotoxic activities of the extract of *Solanum sisymbriifolium* (Lam.) leaves

**Published:** 2013

**Authors:** Apurba Sarker Apu, Shakhawat Hossan Bhuyan, Maima Matin, Faruq Hossain, Farjana Khatun, Abu Taiab, Jamaluddin

**Affiliations:** 1 *Department of Pharmacy, East West University, Plot No-A/2, Main Road, Jahurul Islam City, Aftabnagar, Dhaka-1212, Bangladesh*

**Keywords:** Analgesic, Anti-diarrheal, Anxiolytic, Cytotoxic, Sedative Activity. *Solanum sisymbriifolium*

## Abstract

**Objective**: The present study was undertaken to evaluate the possible analgesic, neuropharmacological, anti-diarrheal, and cytotoxic activities of the ethanol extract of leaves of *Solanum sisymbriifolium* Lam. (Family: Solanaceae).

**Materials and Methods**: The analgesic activity was measured by acetic acid-induced writhing inhibition test. The neuropharmacological activities were evaluated using hole cross, hole board, and elevated plus-maze test and the anti-diarrheal activity was assessed using castor oil-induced diarrhea inhibition method. Brine shrimp lethality bioassay was carried out for assessing the cytotoxicity of the ethanol extract of the leaves. Except cytotoxic activity, all the tests were conducted on mice.

**Results**: The extract at oral doses of 200 and 400 mg/kg body weight showed highly significant (p<0.001) decrease in number of writhing, 52.1±0.66 and 4.4±0.64 compared with the control (78.6±0.29) with the percentage of inhibitions of writhing response were found to be 33.72% and 94.40%, respectively. Compare with the control, the extract at both doses showed significant sedative effect in hole cross test. In hole board test, the extract exhibited highly significant (p<0.001) anxiolytic activity at dose of (200 mg/kg), while the same activity was observed at dose of 400 mg/kg in elevated plus-maze test. The extract showed highly significant (p<0.001) anti-diarrheal activity in a dose-dependent manner. With the extract, significant lethality to brine shrimp was found with LC_50_ value of 61.66±0.9 μg/ml, which was comparable with the positive control (LC_50_: 11.89±0.8 µg/ml).

**Conclusion**: The results from the present studies support the traditional uses of this plant part and could form the basis of further investigation including compound isolation.

## Introduction

*Solanum sisymbriifolium *Lam. (Family: Solanaceae) is a viscoid and very prickly erect shrub commonly known as Kanta begun, Kantikari (Bengali), Sticky nightshade (English), etc. Its fruits are globose-obovoid and shiny red when ripe, 0.5-1.5 cm in diameter. *Solanum sisymbriifolium* (*S*.* sisymbriifolium*) is native to South America. However, it is also cultivated in North America, Europe, Asia, Africa, and Australasia (Chakravarty et al., 1996 ▶). The chemical constituents previously reported to be found in roots of *S. sisymbriifolium* were cuscohygrine, solacaproine (Ferro et al., 2005 ▶), and isonuatigenin-3-*O*-β-solatriose (Ibarrola et al., 2000 ▶). The berries contain sisymbriifolin (a neolignan) and carpesterol (a rare C_30 _sterol) (Chakravarty et al., 1996 ▶). The leaves of *S. sisymbriifolium* are traditionally being used as febrifuge in Peru and diuretic in Brazil, while its roots are used as diuretic, analgesic, hysteria, contraceptive, antisyphilitic, and hepatoprotective in Argentina (Ferro et al., 2005 ▶). The fruits and flowers are used as analgesic in India and in the synthesis of corticosteroids and oral contraceptives (Ferro et al., 2005 ▶). No scientific references on any experimental evaluation of the analgesic, neuropharmacological, anti-diarrheal, and cytotoxic activities have been found which traditional medicine ascribes to this plant. Therefore, to establish its traditional uses, the present investigations were carried out to study the pharmacological activities of ethanol extract of leaves of *S. sisymbriifolium* available in Bangladesh.

## Materials and Methods

**Plant materials**

The fresh leaves of *S. sisymbriifolium *were collected from Agargaon, Dhaka in the month of August, 2011, and were identified by a taxonomist (Dr. Bushra Khan, Principal Scientific Officer) of Bangladesh National Herbarium, Mirpur, Dhaka as *S. sisymbriifolium *Lam. A voucher specimen (Accession No. DACB 35894) for *S*.* sisymbriifolium* has been deposited for further reference. 

**Preparation of ethanol extract**

The collected leaves of *S. sisymbriifolium* were thoroughly washed with water and sun-dried for 15 days. The dried leaves were pulverized with a locally fabricated grinding machine and were stored in an airtight container. The dried and coarsely powdered plant material (1.5 kg) was extracted by cold extraction process using 99.8% (v/v) ethanol as solvent, through occasional shaking and stirring for 7 days. The solvent was filtered and evaporated (temperature 50 °C, lower pressure, rpm 120) using rotary evaporator (IKA, Germany) to get dried extract (11.6% w/w). The dry extract was kept in a refrigerator until use.

**Experimental animals**

Fifty Swiss albino mice (20-25 g) of either sex were purchased from the Animal Research Branch of the International Center for Diarrheal Disease and Research, Bangladesh (ICDDR, B). The animals were kept under standard laboratory conditions (relative humidity 55-65%, room temperature 25.0±2 °C and 12 hr light/dark cycle) for two weeks prior to experimentation for adaptation with the laboratory conditions. Animals were fasted overnight with free access to water prior to each experiment and the time interval between the tests was two weeks. The mice were fed with standard food (ICDDR, B formulated) and water *ad libitum*. The experimental protocols were approved by the Animal Experimentation Ethics Committee (AEEC) of East West University.

**Analgesic activity**

The peripheral analgesic activity of the extract was evaluated using acetic acid-induced writhing inhibition method in mice (Al-Amin et al., 2011 ▶; Zulfiker et al., 2010 ▶). In this method, mice were randomly divided into four groups, each consisting of five animals. The control group received 1% v/v tween-80 (Merck, Germany) in normal saline (Beximco Infusions Ltd., Bangladesh) at a dose of 0.5 ml/mice. The test groups were treated with the extract at a dose of 200 mg and 400 mg/kg body weight, while the positive control group received diclofenac sodium (Square Pharmaceuticals Ltd., Bangladesh) at dose of 10 mg/kg. The control and test samples were administered orally to the respective groups 30 min (Al-Amin et al., 2011 ▶; Zulfiker et al., 2010 ▶) prior to intraperitoneal administration of 0.7% v/v acetic acid (Merck, Germany) solution (0.1 ml/10 gm). The positive control was administered 15 min prior to the administration of acetic acid. 

The number of writhings (painful muscular contraction) of each mouse was counted individually for a period of 20 min, just 5 min after the administration of acetic acid. Full writhing was not always accomplished by the animals; the animals started to give writhing but they did not complete it. This incomplete writhing was considered as half-writhing. Accordingly, two half-writhings were taken as one full writhing (Al-Amin et al., 2011 ▶; Zulfiker et al., 2010 ▶). Analgesic activity was expressed as writhing inhibition (%) and was calculated for each animal using the following formula: Writhing inhibition (%) = {(W_c_ – W_s_)/W_c_} × 100

Where, W_c_ is the mean number of writhings of the control and W_s_ is the mean number of writhings of the test sample.

**Neuropharmacological activities**

Neuropharmacological activities of ethanol extract of *S. sisymbriifolium* leaves were conducted using hole cross, hole board, and elevated plus-maze tests. During every experiment, four groups of mice each containing 5 mice were taken. Each group received a particular treatment:

Group I: Control (1% v/v tween-80 in normal saline, 0.5 ml/mice).

Group II: Positive control (diazepam, Square Pharmaceuticals Ltd., Bangladesh, 1 mg/kg body weight)

Group III: Test sample I (ethanol extract at the dose of 200 mg/kg body weight)

Group IV: Test sample II (ethanol extract at the dose of 400 mg/kg body weight)

*Hole cross test*

The hole cross test, as described by Subhan et al. (2008) ▶ was adopted for screening the sedative effect of the ethanol extract of *S. sisymbriifolium* leaves in mice. A wooden partition having a size of 30×20×14 cm was fixed in the middle of a cage. A hole (diameter 3 cm) was made in the centre of the cage at a height of 7.5 cm. Each mouse was immediately placed in any of the two chambers of the specified instrument after oral administration of the treatments. The number of passages through the hole from one chamber to another was counted on 0, 30, 60, 90, and 120 min for a 3 min test period (Subhan et al., 2008 ▶). 

*Hole board test*

The hole board test is the widely used valid pharmacological method for assessing anxiolytic and/or anxiogenic activity (Takeda et al., 1998 ▶). The test was performed according to the method described by Barua et al. (2012) ▶. The hole board apparatus consisted of a wooden box (40×40×25 cm) with sixteen equidistant holes (diameter 3 cm) evenly distributed on the base of the box. The apparatus was elevated 25 cm above the floor. After 30 min (Barua et al., 2012 ▶) of oral administration of treatments, each mouse was placed individually on the center of the board (facing away from the observer). Latency to the first head dipping and the numbers of head dipping in a period of 5 min were counted.

*Elevated plus-maze test*

The anxiolytic activity of the extract was evaluated using the elevated plus-maze (EPM) test (Thippeswamy et al., 2011 ▶). The EPM test apparatus consisted of two open arms (16×5 cm each) and two enclosed arms (16×5×12 cm each) that were extended from a common central platform (5×5 cm). The maze was elevated to a height of 40 cm above floor level. Thirty minutes (Thippeswamy et al., 2011 ▶) after the oral treatment with the control, diazepam and *S*. *sisymbriifolium* extract, each mouse was placed individually on the central platform facing towards an open arm. 

The numbers of open and enclosed arms entries, plus the time spent in open and enclosed arms, were recorded for a 5-min test period. An entry into an arm was defined when the mouse had all four paws in the arm. For each mouse, total exploratory activity (number of entries in both arms) and other ethologically derived measures such as grooming (itching of the face by the front legs), rearing (vertical movement against the side and/or end of the walls), and stretch-attend postures (exploratory posture in which the body is stretched forward then retracted to the original position without any forward locomotion) were also determined. All types of animal behavior were recorded using a digital video camera located above the maze.

**Anti-diarrheal activity**

Anti-diarrheal activity of the ethanol extract of *S*. *sisymbriifolium* leaves was tested using the model of castor oil-induced diarrhea in mice (Shoba and Thomas, 2011 ▶). According to the method, mice were randomly divided into four groups and fasted overnight before the experiment. Each group received a particular treatment, i.e., control (1% v/v tween-80 in normal saline, 0.5 ml/mice), positive control (loperamide, Square Pharmaceuticals Ltd., Bangladesh, 2 mg/kg body weight), and test samples (200 mg and 400 mg/kg). After 30 min of oral administration of the controls and test samples, 0.2 ml castor oil (BDH Chemicals Ltd., UK) was administered orally to each mouse to induce diarrhea and the mouse was placed in a separate beaker on a filter paper for observation. During an observation period of 3 hr, a number of parameters were recorded: (a) Onset of dry stool, (b) Onset of wet stool, (c) Number of wet stools, (d) Weight of wet stool, and (e) Total weight of fecal output.

**Cytotoxic activity**

The cytotoxic potency of the extract was screened using brine shrimp lethality bioassay (Meyer et al., 1982 ▶). For the assay, *Artemia salina *leaches (brine shrimp eggs) were collected and hatched in a glass tank containing simulated seawater (3.8% NaCl solution, pH 8.4) at a temperature ~37 °C and equipped with constant oxygen supply. Four mg of the extract was dissolved in 200 µl of Dimethylsulfoxide (DMSO; Merck, Germany) and the volume was adjusted to 5 ml using simulated seawater. Therefore, the concentration of the stock solution was 800 µg/ml. A series of solutions of lower concentrations were prepared by serial dilution using simulated seawater. Two and a half ml solution from each of these test solutions were added to pre-marked vials containing 2.5 ml of seawater and 10 shrimp nauplii. Therefore, the final concentrations of samples in the vials ranged from 400 µg/ml to 0.781 µg/ml. 

**Figure 1:**
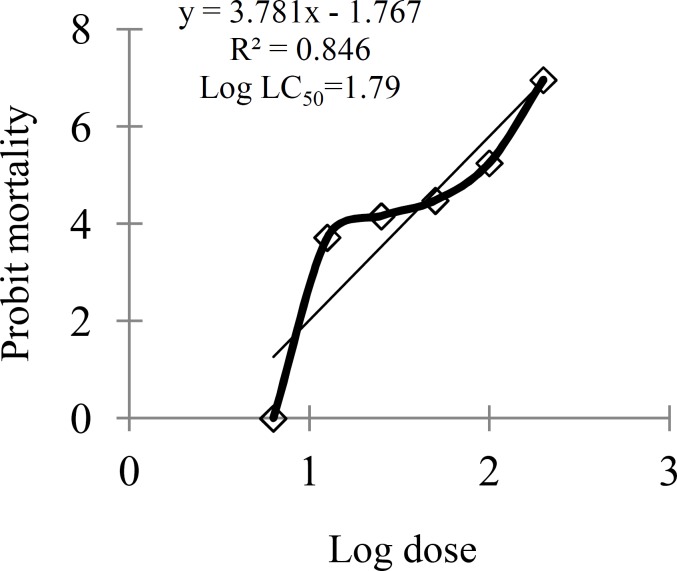
Plot of log doses versus probit for calculation of LC_50_ of ethanol extract of *S*.* sisymbriifolium *leaves. Finney’s statistical method of probit analysis was used for the analysis of the data

In the control vials, same volumes of DMSO (as the sample vials) were taken as negative control while potassium permanganate (KMnO_4_) of different concentration was used as positive control. After 24 hr, the number of active nauplii in each vial was counted and mortality (%) was calculated for each dilution as well as for the control. Percent mortality was corrected and then converted to Probit using Finney’s probit table (Finney, 1971 ▶). After that, the LC_50_ values (concentration of sample required to kill 50% of brine shrimp within 24 hr of exposure) were calculated (at the confidence interval level of 95%) using Microsoft Excel 2007 by a plot of Probit (y) against the logarithm of the sample concentrations (x) (Figure 1).

**Statistical analysis**

All data were presented as mean±SEM. SPSS for WINDOWS^TM^ (version 12.0) was applied for the analysis and the results were statistically analyzed by one-way analysis of variance (ANOVA) followed by Dunnett’s *t*-test (2-sided) to compare the treatments groups with the control group. p<0.05 and p<0.001 were considered to be the level of significance and level of highly significance, respectively.

For brine shrimp lethality test data analysis, Finney’s statistical method of probit analysis (Finney, 1971 ▶) was used to calculate LC_50_ with 95% confidence intervals.

## Results

**Analgesic activity**

In acetic acid-induced writhing inhibition test, the ethanol extract of *S. sisymbriifolium* at both doses (200 and 400 mg/kg body weight) showed highly significant (p<0.001) inhibition of writhing response induced by the acetic acid in a dose-dependent manner. The percentage of inhibitions of the writhing response at the doses 200 mg/kg and 400 mg/kg were 33.72% and 94.40%, respectively (Table 1) which was comparable with the positive control diclofenac sodium (98.09%).

**Table 1:** Effect of ethanol extract of *S. sisymbriifolium* leaves (SSL) and diclofenac sodium on acetic acid induced writhing in mice

**Treatment**	**Dose (p.o.)**	**No. of writhing**	**% inhibition**
**Control**	0.5 ml/mice	78.6±0.29	---
**Diclofenac sodium** a	10 mg/kg	1.5±0.16***	98.09
**SSL200**	200 mg/kg	52.1±0.66***	33.72
**SSL400**	400 mg/kg	4.4±0.64***	94.40

Values are expressed as mean±SEM, n=5,

*** p<0.001 compared with the control; SSL: ethanol extract of *S*. *sisymbriifolium* leaves; SSL200 and SSL400 indicate dose of 200 and 400 mg/kg body weight, respectively;

a Positive control. One-way analysis of variance (ANOVA) followed by Dunnett’s *t*-test (2-sided) was used for the analysis of the data.

**Neuropharmacological activities**

*Hole cross test*

In the hole cross test, compare with the control group, the extract at both doses (200 and 400 mg/kg body weight) showed decrease in locomotion activity in the test animals as evident by the decrease in number of movement from one chamber to another in the cage at all the observation periods except the 3^rd^ observation period (60 min) (Table 2). At the 3^rd^ observation period, an increase in locomotion activity was observed in the mice by the extract at dose of 400 mg/kg body weight but the result was not statistically significant. Treatment with diazepam, the positive control, showed decrease in locomotion activity at all the observation periods (Table 2).

*Hole board test*

In the hole board test, the extract at the dose of 200 mg/kg body weight showed highly significant (p<0.001) increase in the number of head dipping (65.6±0.68) behavior compare with the control group (44.8±1.24) (Table 3). In contrast, the number of head dipping behavior was decreased (35.8±0.80) by treatment with the extract at dose of 400 mg/kg which was statistically highly significant (p<0.001). The positive control (diazepam) treated mice also showed statistically highly significant (p<0.001) decrease in the number of head dips (29.6±0.93) compared with the control group (Table 3).

*Elevated plus-maze test*

In elevated plus-maze (EPM) test, *S. sisymbriifolium* extract-treated mice at dose of 200 mg/kg exhibited (Table 4) highly significant (p<0.001) decrease in the percentage of time spent in the open arms (1.32±0.20) compared with the control group (3.41±0.51), whereas the oral treatment of mice with 400 mg/kg of the extract showed highly significant (p<0.001) increase in the percentage of time spend in open arms (6.55±0.51) (Table 4). The positive control, diazepam, at dose of 1 mg/kg treated mice showed a significant (p<0.05) decrease in the percentage of time spent in the open arms (2.55±0.04) (Table 4).

**Table 2:** Effect of ethanol extract of *S. sisymbriifolium* leaves (SSL) and diazepam on number of movements in hole cross test

**Treatment**	**Dose (p.o.)**	**No. of movements**				
		**0 min**	**30 min**	**60 min**	**90 min**	**120 min**
**Control**	0.5 ml/mice	10.0±0.71	8.6±0.60	7.2±0.58	7.0±0.55	6.2±0.37
**Diazepam** a	1 mg/kg	7.8±0.74	6.2±0.80*	5.8±0.74	4.2±1.16*	3.6±0.51*
**SSL200**	200 mg/kg	5.8±0.37	4.2±0.37***	4.8±0.49	5.8±0.37	6.2±0.74
**SSL400**	400 mg/kg	3.6±0.51	6.2±0.49*	8.0±0.89	4.0±0.63*	3.8±0.49*

Values are expressed as mean±SEM, n=5,

* p<0.05,

*** p<0.001 compared with the control; SSL: ethanol extract of *S. sisymbriifolium* leaves; SSL200 and SSL400 indicate dose of 200 and 400 mg/kg body weight, respectively;

a Positive control. One-way analysis of variance (ANOVA) followed by Dunnett’s *t*-test (2-sided) was used for the analysis of the data.

**Table 3:** Effect of ethanol extract of *S. sisymbriifolium* leaves (SSL) and diazepam in hole board test in mice

**Treatment**	**Dose (p.o.)**	**No. of head dipping**	**Latency to the first head dipping (sec)**
**Control**	0.5 ml/mice	44.8±1.24	14.8±0.37
**Diazepam** a	1 mg/kg	29.6±0.93***	2.0±0.45***
**SSL200**	200 mg/kg	65.6±0.68***	16.2±0.66
**SSL400**	400 mg/kg	35.8±0.80***	6.4±0.40***

Values are expressed as mean±SEM, n=5,

*** p<0.001 compared to control; SSL: ethanol extract of *S. sisymbriifolium* leaves; SSL200 and SSL400 indicate dose of 200 and 400 mg/kg body weight, respectively;

a Positive control. One-way analysis of variance (ANOVA) followed by Dunnett’s *t*-test (2-sided) was used for the analysis of the data.

**Table 4:** Effect of ethanol extract of *S. sisymbriifolium* leaves (SSL) and diazepam on behavior of mice in elevated plus-maze model test.

**Treatment**	**Dose** **(p.o.)**	**Time (sec) spent in**		**No. of entry in**		**% of total time (sec) spent in**		**% of entries in**	
		**Open** **arm**	**Close** **arm**	**Open** **arm**	**Close** **arm**	**Open** **arm**	**Close** **arm**	**Open** **arm**	**Close** **arm**
**Control**	0.5 ml/mice	7.6±0.51	215±1.52	1.2±0.49	9.4±0.51	3.41±0.05	96.59±0.14	11.32±0.49	88.68±0.51
**Diazepam** a	1 mg/kg	6.8±0.58	260±1.87***	1.6±0.81	12.0±0.55*	2.55±0.04*	97.45±0.14	11.76±0.81	88.24±0.55
**SSL200**	200 mg/kg	2.8±0.20***	209±1.44	0.8±0.37	15.4±0.75***	1.32±0.02***	98.68±0.14	4.94±0.37	95.06±0.75
**SSL400**	400 mg/kg	16.6±0.51***	237±1.95***	0.8±0.37	9.0±0.45	6.55±0.04***	93.45±0.15	8.16±0.37	91.84±0.45

Values are expressed as mean±SEM, *n*=5,

* p<0.05,

*** p<0.001 compared to control; SSL: ethanol extract of *S. sisymbriifolium* leaves; SSL200 and SSL400 indicate dose of 200 and 400 mg/kg body weight, respectively;

a Positive control. One-way analysis of variance (ANOVA) followed by Dunnett’s *t*-test (2-sided) was used for the analysis of the data.

**Figure 2:**
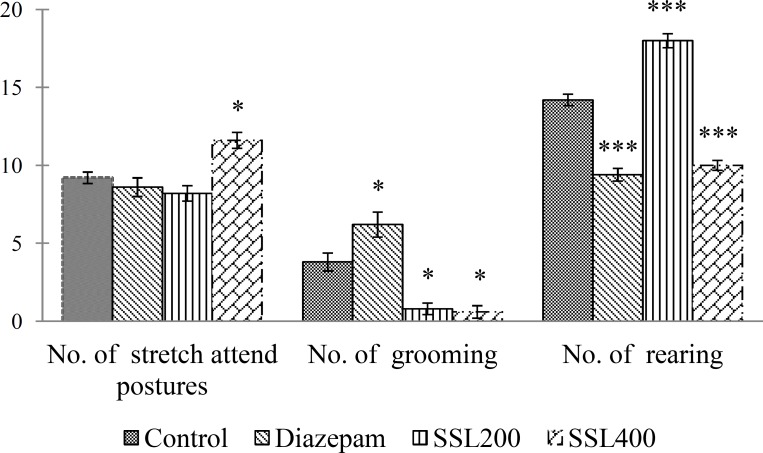
Effect of diazepam and ethanol extract of *S*. *sisymbriifolium* leaves (SSL) on ethologically derived measures in mice tested on the elevated plus-maze.

The overall effects of the oral administration of the extract on mice, ethologically derived measures are shown in Figure 2. Statistical analysis revealed that the extract at dose of 400 mg/kg induced a significant decrease in grooming (p<0.05) and rearing behavior (p<0.001) compare with the control. However, a highly significant (p<0.001) increase in rearing behavior was observed by treatment with the extract at dose of 200 mg/kg (Figure 2).

**Anti-diarrheal activity**

In castor oil-induced diarrhea inhibition test, *S. sisymbriifolium* leaves (SSL) extract at the dose of 200 mg/kg body weight showed (Table 5) an increase in mean latent period for diarrhea episode (56.8±1.24 min), whereas at the dose of 400 mg/kg showed a decrease in mean latent period for diarrhea episode (7.2±0.86 min) compared with the control. However, the extract at both doses showed significant (*p*<0.05) decrease in mean number of stools and total weight of fecal output in a dose-dependent manner (Table 5).

**Cytotoxic activity**

In brine shrimp lethality bioassay, varying degrees of lethality were observed with exposure to different dose levels of the test samples. The percentage of mortality increased gradually with the increase in concentration of the test samples. In comparison, the LC_50_ value of the ethanol extract of *S*. *sisymbriifolium* leaves was found to be 61.66±0.9 g/ml (Table 6) whereas the positive control (KMnO_4_) showed LC_50 _value of 11.89±0.8 g/ml (Table 6).

**Table 5:** Effect of ethanol extract of *S. sisymbriifolium* leaves (SSL) and loperamide on castor oil-induced diarrhea in mice

**Treatment**	**Dose (p.o.)**	**Latent period (min)**	**No. of stools**	**Total weight of fecal output**
**Control**	0.5 ml/mice	42.8±1.16	14.4±1.75	0.935±0.02
**Loperamide** a	2 mg/kg	95.2±1.71***	8.6±0.75*	0.731±0.03*
**SSL200**	200 mg/kg	56.8±1.24***	9.6±0.81*	0.768±0.04*
**SSL400**	400 mg/kg	7.2±0.86***	6.6±0.75***	0.731±0.03***

Values are expressed as mean±SEM, n=5,

* p<0.05,

*** p<0.001 compared to control; SSL: ethanol extract of *S. sisymbriifolium* leaves; SSL200 and SSL400 indicate dose of 200 and 400 mg/kg body weight respectively;

a Positive control. One-way analysis of variance (ANOVA) followed by Dunnett’s *t*-test (2-sided) was used for the analysis of the data.

**Table 6:** Cytotoxic activity of ethanol extract of *S. sisymbriifolium* leaves (SSL)^.^

**Plant**	**Part** **used**	**Tested** **materials**	**LC**_50_ **(µg/ml, 24 h)**	**95% CI ** **(µg/ml)**	**Linear regression** **equation**	**R** ^2^
***Solanum sisymbriifolium***	Leaves	Ethanol extract	61.66±0.9	59.90 – 63.43	y = 3.781x - 1.767	0.846
		KMnO_4_^a^	11.89±0.8	11.73 – 12.06	y = 5.181x - 0.572	0.851

Values are expressed as mean±SEM*, *n=3,

a Reference standard; SSL: ethanol extract of *S. sisymbriifolium* leaves. Finney’s statistical method of probit analysis was used for the analysis of data.

## Discussion

**Analgesic activity**

In the analgesic activity performed using acetic acid-induced writhing inhibition test in mice, the writhing inhibition increased as the dose of extract was increased (Table 1). Preliminary qualitative phytochemical screening (Shilpi et al., 2005 ▶) reveals the presence of alkaloids, flavonoids, steroids, and tannins in *S. sisymbriifolium*. Flavonoids, tannins, and alkaloids have been reported to have a role in analgesic activity primarily by targeting prostaglandins (Rahman, 2012 ▶; Zulfiker et al., 2010 ▶). The presence of steroids may also partly contribute to the analgesic effect of the extract (Rahman, 2012 ▶). Findings in this study justify the traditional use of *S. sisymbriifolium *as an analgesic in India (Ferro et al., 2005 ▶). 

**Neuropharmacological activities**

*Hole cross test*

Gamma-amino-butyric acid (GABA) is the major inhibitory neurotransmitter in the central nervous system. CNS depressant drugs mainly exert their action through GABA_A_ receptor (Dolai et al., 2012 ▶). Therefore, the sedative effect of the extract at both doses (200 and 400 mg/kg body weight, Table 2) may be due to hyperpolarization of the CNS via interaction with GABA_A_ or benzodiazepine receptor. Further studies are needed to evaluate this. In addition to the sedative effect, the decrease in movement may be due to the muscle relaxant effect of the plant extract. The decrease in locomotion activity by diazepam treated mice compare with the control may be due to the dose (1 mg/kg) used in the test that can produce sedation in mice as reported by Takeda et al. (1998) ▶. 

*Hole board test*

Takeda et al. (1998) ▶ reported that a decrease in head dipping behavior in hole board test reflects the anxiogenic state of animals, while an increase in head dipping behavior reflects anxiolytic state. Based on the report, the result (Table 3) of the present study demonstrates that the ethanol extract of *S. sisymbriifolium* leaves at dose of 200 and 400 mg/kg body weight possesses anxiolytic and anxiogenic activity, respectively. Besides the observed anxiogenic effect at dose of 400 mg/kg, the decrease in number of head dips also reveals sedative behavior (File and Wardill, 1975 ▶) and is thus a measure of CNS depressant activity of the plant extract (Viswanatha et al., 2006 ▶). Earlier reports on the chemical constituents of plants and their pharmacology suggest that the plant containing flavonoids and tannins possesses activity against many CNS disorders (Adeyemi et al., 2006 ▶). Therefore, it is possible that the mechanism of anxiolytic effects of the extract in hole board test at the lower dose (200 mg/kg) may be due to the binding of any of the phytochemicals to the GABA_A_-benzodiazepines (BZDs) complex; however, further studies are needed to ascertain this. Moreover, it is possible that the observed sedative activity of the extract at higher dose (400 mg/kg) may be mediated by GABAergic pathway, since the deep sedation in mice can be produced by GABAergic transmission (Gottesmann 2002 ▶). Further detailed investigations are needed to be conducted to establish the actual mechanism(s) by which the extract showed the observed activity. The highly significant (p<0.001) decrease in head dipping behavior by diazepam compare with the control may be due to the sedative dose (1 mg/kg) used in the test (Takeda et al., 1998 ▶). 

*Elevated plus-maze test*

In elevated plus-maze (EPM) test, the extract at dose of 400 mg/kg body weight showed anxiolytic activity (Table 4) compare with the control which was observed at dose of 200 mg/kg in hole board test (Table 3). Adamec and Shallow (2000) ▶ reported that the time interval of less than 3 weeks between hole board and elevated plus-maze tests results in open arm avoidance in EPM test. In the present study, the time interval between these two tests was 2 weeks. Therefore, the observed controversial results found in EPM test may be due to the short time interval between the tests.

Plants containing sterols, flavonoids, saponins, and tannins are reported to have anxiolytic activity (Gadekar et al., 2011 ▶) and preliminary phytochemical screening revealed the presence of sterols, tannins, and flavonoids in *S. sisymbriifolium*. Therefore, the anxiolytic activity of the extract at 400 mg/kg body weight may be due to the binding of any of these phytochemicals to the GABA_A_-BZDs complex, which requires further study.

**Anti-diarrheal activity**

On the basis of the result (Table 5) of castor oil-induced diarrhea inhibition test, it is shown that the ethanol extract of* S. sisymbriifolium* leaves might possess significant anti-diarrheal activity, which was evident from the decreased number of stools as well as the decrease in total weight of fecal output. Castor oil develops diarrhea through the stimulation of peristaltic activity and synthesis of prostaglandin and also by preventing the reabsorption of water in the small intestine (Bose et al., 2012 ▶). Okudo et al. (1989) ▶ reported that the tannins can induce anti-diarrheal effect through the precipitation of proteins of the enterocytes; reduction of peristaltic movement and intestinal secretions. On the other hand, flavonoids are also reported to possess anti-diarrheal activity due to their ability to inhibit enzymatic metabolism of arachidonic acid, hydro-electrolytic secretions, intestinal motility, PGE_2_ induced intestinal secretory response (Bose et al., 2012 ▶). Preliminary qualitative phytochemical screening (Shilpi et al., 2005 ▶) of *S. sisymbriifolium* reveals that the plant is a rich source of alkaloids, flavonoids, steroids, and tannins which justify the significant anti-diarrheal effect of the plant extract.

**Cytotoxic activity**

The plant extract is considered to be significantly cytotoxic when LC_50_ value obtained in brine shrimp lethality test is 250 μg/ml or less (Apu et al., 2010 ▶). Therefore, the result (Table 6) of the present study clearly indicates that the plant is significantly cytotoxic. Moreover, cytotoxic plant also possesses a wide range of pharmacological activities, such as antimicrobial, pesticidal, and antitumor activities (Anderson et al., 1988 ▶) and therefore the positive response obtained in this bioassay demands further investigation of *S. sisymbriifolium* leaves.
